# Edible Scaffolds Based on Non-Mammalian Biopolymers for Myoblast Growth

**DOI:** 10.3390/ma10121404

**Published:** 2017-12-08

**Authors:** Javier Enrione, Jonny J. Blaker, Donald I. Brown, Caroline R. Weinstein-Oppenheimer, Marzena Pepczynska, Yusser Olguín, Elizabeth Sánchez, Cristian A. Acevedo

**Affiliations:** 1 Biopolymer Research and Engineering Lab (BiopREL), Universidad de los Andes, Avenida Monseñor Alvaro del Portillo 12455, Las Condes, Santiago 7550000, Chile; jenrione@uandes.cl (J.E.); mpepczynska@uandes.cl (M.P.); 2 Bio-Active Materials Group, School of Materials, MSS Tower, The University of Manchester, Manchester M13 9PL, UK; jonny.blaker@manchester.ac.uk; 3 Laboratorio de Biología de la Reproducción y del Desarrollo, Instituto de Biología, Facultad de Ciencias, Universidad de Valparaíso, Avenida Gran Bretaña 1111, Valparaíso 2340000, Chile; donald.brown@uv.cl; 4 Escuela de Química y Farmacia, Facultad de Farmacia, Universidad de Valparaíso, Avenida Gran Bretaña 1093, Valparaíso 2340000, Chile; caroline.weinstein@uv.cl; 5 Center for Integrative Medicine and Innovative Science (CIMIS), Universidad Andrés Bello, Echaurren 183, Santiago 8320000, Chile; yusser.olguin@unab.cl; 6 Centro de Biotecnología, Universidad Técnica Federico Santa María, Avenida España 1680, Valparaíso 2340000, Chile; elizabeth.sanchez@usm.cl; 7 Departamento de Física, Universidad Técnica Federico Santa María, Avenida España 1680, Valparaíso 2340000, Chile

**Keywords:** biopolymer, edible material, in vitro meat, scaffold

## Abstract

In vitro meat has recently emerged as a new concept in food biotechnology. Methods to produce in vitro meat generally involve the growth of muscle cells that are cultured on scaffolds using bioreactors. Suitable scaffold design and manufacture are critical to downstream culture and meat production. Most current scaffolds are based on mammalian-derived biomaterials, the use of which is counter to the desire to obviate mammal slaughter in artificial meat production. Consequently, most of the knowledge is related to the design and control of scaffold properties based on these mammalian-sourced materials. To address this, four different scaffold materials were formulated using non-mammalian sources, namely, salmon gelatin, alginate, and additives including gelling agents and plasticizers. The scaffolds were produced using a freeze-drying process, and the physical, mechanical, and biological properties of the scaffolds were evaluated. The most promising scaffolds were produced from salmon gelatin, alginate, agarose, and glycerol, which exhibited relatively large pore sizes (~200 μm diameter) and biocompatibility, permitting myoblast cell adhesion (~40%) and growth (~24 h duplication time). The biodegradation profiles of the scaffolds were followed, and were observed to be less than 25% after 4 weeks. The scaffolds enabled suitable myogenic response, with high cell proliferation, viability, and adequate cell distribution throughout. This system composed of non-mammalian edible scaffold material and muscle-cells is promising for the production of in vitro meat.

## 1. Introduction

In vitro meat is an emerging technology that involves the production of edible muscle tissue in a laboratory setting, and the use of biotechnological tools for the production of synthetic tissues. Tissue engineering of skin, cartilage, and bone have been extensively developed [1,2,3], as has the engineering of skeletal muscle [4]. While the main application of these synthetic tissues has been for biomedical uses (e.g., tissue repair and regeneration), these techniques can be applied to produce meat, avoiding the slaughter of farmed animals. There are numerous publications that discuss the potential benefits of manufacturing in vitro meat or cultured beef in the future [5,6,7,8,9,10]. Some of these benefits are related to animal welfare, improving human health (e.g., transmissible spongiform encephalopathy), food hazards (e.g., salmonellosis), and reducing environmental impacts (e.g., global warming) [8].

Methods to produce in vitro meat commonly employ the growth of myoblasts on a scaffold suspended in culture medium within a bioreactor [6]. The principal goals that form the basis for this technology are: (i) the culture of muscle progenitor cells without the need to slaughter the animal; (ii) the design of edible scaffolds that are suitable for myoblast proliferation; (iii) the formulation of serum-free cell culture media; (iv) the use of bioreactors where myogenic stimuli can be applied to obtain muscle fibers. This work aims to contribute to the second goal, in developing and evaluating an edible three-dimensional (3D) porous construct (scaffold) that does not contain materials sourced from mammals.

Here, the scaffold is defined as a porous material where the anchorage-dependent cells (e.g., muscle cells) can remain viable and proliferate. The scaffold must be biocompatible, and must have appropriate microstructure and physical properties to enable cell attachment and proliferation [11]. Pore size and stiffness are important scaffold design parameters for skin, bone, nerve, and muscle tissues [11,12]. Specifically for muscle cell culture, the selection of soft porous materials with adequate microstructure and stiffness are important [12].

Myoblasts have been shown to remain viable when cultured on non-edible commercial materials such as Matrigel^®^ [13], however to the author’s knowledge there are no commercially available edible scaffolds for in vitro meat production. Though there were previous reports on scaffolds for tissue engineering, there are disadvantages such as the use of non-edible crosslinkers such as carbodiimide (EDC) or glutaraldehyde [1,2,3,11]. A further significant issue is that the available scaffolds are frequently based on mammalian biomaterials. There is extensive literature concerning the use of bovine gelatin, collagen, fibrin, hyaluronic acid, and other biopolymers that show remarkable results in the field of tissue engineering [1,14,15]. A key aim of in vitro meat synthesis is to obviate mammal slaughter, and therefore mammalian-derived biomaterials should be avoided.

Non-mammalian biopolymers extracted from algae (e.g., alginate and agar) or fish species (gelatin) have been used in tissue engineering [16,17,18]. However, while alginate and agar permit the culture of mammalian cells [18,19], they do not contain cell recognition cites—the Arg-Gly-Asp (RGD) sequences that promote cell adhesion and migration [20]. As gelatin contains RGD sequences, a promising strategy is to blend algae-derived polymers with fish-derived gelatin. Salmon gelatin is an attractive ingredient to prepare such edible and biodegradable scaffolds [21]. In addition, due to its physical properties (and lower melting temperature than other mammalian gelatin sources), salmon gelatin can be easily blended with other biopolymers, allowing the formation of copolymers and stable polyelectrolyte complexes [22].

Since myoblast cell proliferation is the first step in producing muscle fibers ahead of myogenic stimulation, we hypothesize that edible materials based on salmon gelatin, modified with other biopolymers (alginate, agar, and agarose), can be used as 3D scaffolds for the cultivation of myoblasts. Here, edible scaffolds are formulated using non-mammalian biopolymers, biological active protein from salmon gelatin, combined with alginate (cross-linked by calcium alginate), and gelling agents (agar and agarose).

## 2. Materials and Methods

### 2.1. Experimental Strategy

Solutions of salmon gelatin (cationic) and alginate (anionic) were first prepared to form a stable polyelectrolyte complex without precipitation of the components; these were then converted into stable hydrogels by the incorporation of gelling agents and plasticizers. These hydrogels were then frozen and subsequently freeze-dried to yield 3D porous scaffolds.

**Figure 1:**
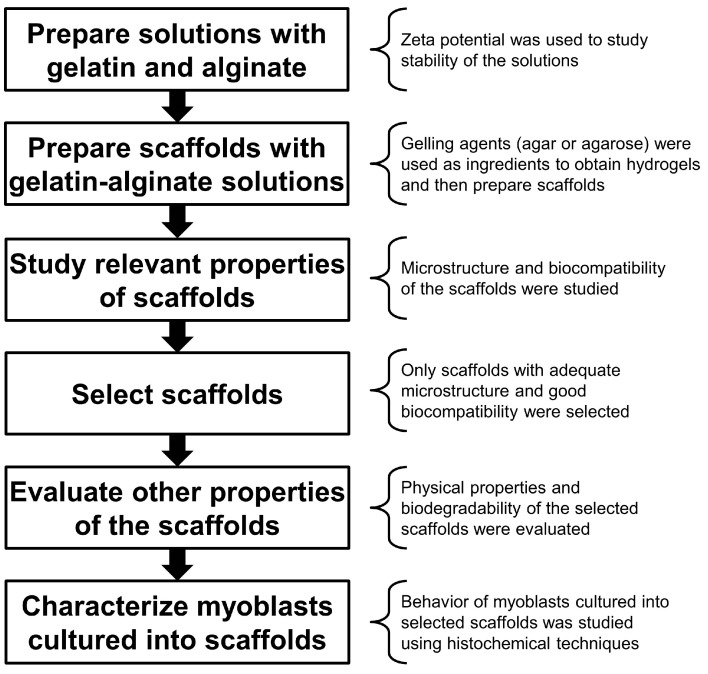
Experimental strategy.

The microstructural and physical properties, as well as biological response, were then characterized, via myoblast culture and histochemical techniques. The experimental strategy followed in this study is outlined in Figure 1.

### 2.2. Salmon Gelatin

Salmon gelatin was extracted from salmon skins (*Salmo salar*) following the method proposed by Zhou and Regenstein [23], modified by Díaz et al. [24], and used by Acevedo et al. [22] to produce edible materials.

### 2.3. Evaluation of Zeta Potential of the Biopolymer Solutions

The stability of solutions composed of salmon gelatin and sodium alginate (Food grade, Loba Chemie, Mumbai, India) was assessed using Zeta potential measurements of dilute aqueous solutions (0.1% *w*/*v*). The gelatin:alginate proportions were varied at 0:10, 1:9, 2:8, 3:7, 4:6, 5:5, 6:4, 7:3, 8:2, 9:1, and 10:0.

Zeta potential was measured with a Laser Doppler Velocimetry device (Zetasizer Nano ZS90, Malvern, UK) using previously reported standard procedures [25,26]. 

### 2.4. Scaffold Preparation

Cationic (salmon gelatin) and anionic (sodium alginate) biopolymers were blended to form stable solutions. Subsequently, plasticizer (glycerol, Merck, Darmstadt, Germany) and gelling agent (agar or agarose, Loba Chemie, Mumbai, India) were added to form stable hydrogels. These gels were then frozen to induce phase separation, and subsequently freeze-dried to sublime the ice.

Typically, to obtain 100 mL of the hydrogel polymer solution, a volume of 25 mL of salmon gelatin aqueous solution (1.5% *w*/*v*, gelatin to water) was mixed with 25 mL of sodium alginate aqueous solution (1.5% *w*/*v*) using gentle agitation at 50 °C for 1 h. Subsequently, 50 mL of a diluted excipient solution containing 0.5% *w*/*v* of gelling agent (agar or agarose) with or without plasticizer (0.2% *w*/*v* of glycerol) was added gently and blended by agitation at 50 °C for 1 h. The compositions of the four hydrogel polymer solutions are given in Table 1.

These solutions were poured into a Petri dish, adjusting the volume to obtain a height of 3 mm. These were then cooled at 4 °C to induce gelation, then transferred to a −20 °C freezer overnight and then transferred to a −80 °C freezer for 24 h prior to lyophilization. Porous scaffolds were produced by lyophilization (using a Liobras L101 freeze-drier, Sao Carlos, Brazil), and the scaffolds were stored in a dry environment with silica gel prior to further analysis, detailed below.

Prior to cell seeding, the scaffolds were soaked in CaCl_2_ solution (70 mM) for 1 h at room temperature. This step was performed to prevent the deformation of the porous scaffolds and to keep their structure. Calcium allows the crosslinking of the sodium alginate present in the scaffold to obtain an insoluble hydrogel. Then, the scaffolds were washed three times with sterile water, and then in 70% ethanol for 3 h to sterilize the scaffolds, followed by three further rinses with sterile water.

### 2.5. Scaffold Microstructural Characterization

The scaffold morphology and microstructure were assessed using scanning electron microscopy (SEM). Sectioned scaffold samples were mounted and gold-coated (10–20 nm thickness) by using diode magnetron sputtering equipment (SPI Sputter Coater model 12161, West Chester, PA, USA). Coated samples were examined with a Carl Zeiss SEM (EVO MA 10, Oberkochen, Germany) using 25 kV acceleration voltages.

The pore size (equivalent circular diameter) of the scaffolds was determined from the SEM images using ImageJ Software (NIH, version 1.51k, Bethesda, MD, USA), with at least 100 pores per scaffold measured [27].

### 2.6. Myoblast Cell Culture

The myoblasts cell line C2C12 was used as model of muscle-cells, purchased from the European Collection Cell Cultures (ECACC), supplied by Sigma-Aldrich (St. Louis, MO, USA). C2C12 are immortalized mouse myogenic cell line derived from satellite cells, whose behavior corresponds to that of the progenitor lineage. These cells are a subclone of C2 myoblasts that are known to differentiate into muscle fibers [28].

C2C12 myoblast cells were cultured using standard techniques for cell culture, as follows. Cells were cultured at standard conditions (37 °C and 5% CO_2_ in a humidified atmosphere) using DMEM as cell culture medium (Gibco, Life Technologies, Grand Island, NY, USA), supplemented with 10% fetal bovine serum (Biologicals Industries, Kibbutz Beit-Haemek, Israel), l-glutamine (2 mM), and antibiotics (100 U/mL of penicillin and 100 µg/mL of streptomycin).

### 2.7. Evaluation of Scaffold Biocompatibility

The capacity of the myoblasts to adhere onto the scaffolds and grow into the scaffold was used to indicate cell–scaffold compatibility. Myoblasts were seeded onto the scaffolds at 1 × 10^4^ cells/cm^2^ (scaffold thickness ~3 mm). Then, they were incubated in 24-well culture plates with 800 µL of culture medium at 37 °C and 5% CO_2_. Cell adhesion and growth were determined by estimation of the viable biomass at different times by using the resazurin assay.

Cell adhesion was assessed after 4 h incubation and determined as the quotient of adhered cells at 4 h and loaded cells. Cell growth was determined at 24, 48, and 72 h; at each sampling time, the scaffold was gently removed and the viable cells into the scaffold was measured with the resazurin assay [11], as described below.

Scaffolds with cells were incubated in fresh medium with resazurin (4 mg/L, Sigma-Aldrich, St. Louis, MO, USA) for 4 h at 37 °C. The viable cell numbers were estimated by resorufin production, determined by fluorescence (excitation at 544 nm and emission at 590 nm) with a plate reader (Appliskan, Thermo Fisher Scientific, Vantaa, Finland). For each experiment, a calibration of a known viable cell number was made (cells were counted in a Neubauer chamber with the viability dye trypan blue).

### 2.8. Determination of the Scaffold Moisture Sorption Isotherm

Moisture sorption isotherms of the scaffolds were determined by dynamic vapor sorption (DVS; DVS-INTRINSIC, Surface Measurement Systems Ltd., London, UK).

Approximately 10 mg of each scaffold was weighed into the DVS sample chamber and dried under nitrogen flow (0% relative humidity, RH) until equilibrium (no weight change). Samples were then subjected to a sorption cycle using 10% RH increments between 0% and 80% RH at 20 °C. Equilibrium mass at each RH was determined with a value dm/dt = 0.002% min^−1^. 

The monolayer moisture content was calculated for each scaffold by fitting the data to Guggenheim–Anderson–de Boer (GAB) model [27].

### 2.9. Scaffold Water Uptake Evaluation

Scaffolds were cut in sections measuring 2.5 cm diameter (~5 cm^2^), and their dry weights were recorded (as W_D_). Then, the samples were soaked for 1 h in an aqueous solution of CaCl_2_ (70 mM concentration). Scaffolds were then rinsed three times with distilled water left immersed in water overnight. The scaffolds were removed and blotted with filter paper to remove excess water and their wet weight (W_W_) was recorded. The water uptake was determined as (W_W_ − W_D_)/W_D_.

### 2.10. Scaffold Mechanical Behavior by Dynamic Mechanical Analysis

Wet scaffolds were assessed using a Dynamic Mechanical Analyzer (DMA 1 Star System, Mettler-Toledo, Greifensee, Switzerland) equipped with a liquid nitrogen cooling device, in compression mode. The scaffolds were soaked 1 h in a solution of CaCl_2_ 70 mM, washed three times with distilled water, and blotted to remove excess liquid. Applied oscillatory frequencies were 1, 10, 25, and 40 Hz with a temperature regime of 0 to 50 °C at 3 °C/min. The storage modulus (E’) was obtained across this temperature range.

### 2.11. Evaluation of Scaffold Biodegradation

The biodegradation kinetics of the scaffolds in the presence of lysozyme was determined by an in vitro assay using a gravimetric method [29]. Lysozyme from chicken egg white was purchased from Sigma-Aldrich (St. Louis, MO, USA). The scaffolds were crosslinked using a 70 mM CaCl_2_ solution for 1 h, washed three times with distilled water, and incubated in a lysozyme solution (100 µg/mL) at 37 °C. The initial dry weight of the scaffold was noted as W_I_. The dry weight of the samples was determined by drying the material at 105 °C overnight. The scaffolds incubated with lysozyme at 37 °C were sampled after 1, 2, 3, and 4 weeks. The dry weight of the degraded sample was noted as W_F_, and the percentage of degradation was calculated as 100·(W_I_ − W_F_)/W_I_.

### 2.12. Characterization of Cells Cultured within Scaffolds via Histochemical and Immunohostochemical Tools

Myoblasts were loaded onto the scaffolds at 1 × 10^5^ cells/cm^2^ (scaffold thickness ~3 mm), and cultured in 24-well plates for 2 days at 37 °C and 5% CO_2_. The scaffolds were then fixed in Bouin’s solution for 24 h at 5 °C. Subsequently, the scaffolds were dehydrated and embedded in Paraplast-Plus (Sigma-Aldrich, St. Louis, MO, USA) for histological preparation and cut completely through their thickness (circa 3 mm) using a microtome (Leica, Wetzlar, Germany). Serial sections of 5 μm were obtained and mounted on silane-coated microscope slides, de-paraffinized, and rehydrated ahead of staining.

Cell morphology and distribution were determined using a histochemical trichrome stain (hematoxylin/erythrosine B-orange G/methyl blue) [11]. The first section of each series (15 sections) was stained and analyzed. Cell viability was quantified as the ratio between viable cells (non-pyknotic cells) and total cells. 

Mitotic cells were identified by immunohistochemistry. Prior to fixing, the cells on the scaffolds were cultured for 4 h with a cell culture medium containing BrdU (1% commercial labeling reagent with BrdU; Zymed, South San Francisco, CA, USA). Subsequently, an anti-BrdU biotinylated antibody (dilution 1:500; mouse monoclonal ZBU30; Invitrogen, Camarillo, CA, USA) was applied according to the manufacturer’s instructions. The amplification was performed using the immuno-peroxidase technique using a commercial kit of avidin–biotin–peroxidase complex (ABC) (Vectastain-ABC, Vector, Burlingame, CA, USA) and diaminobenzidine (Sigma-Aldrich, St. Louis, MO, USA), as chromogen, which produces a brown color for positive detection.

Optical microscopy was performed using a Leica DM2500 microscope (Leica Microsystems, Wetzlar, Germany), and the photomicrographs were obtained using a Leitz DMRBE microscope (Leica Microsystems, Wetzlar, Germany) equipped with a DFC290 digital camera (Leica Microsystems, Wetzlar, Germany).

### 2.13. Statistical Analysis

Experiments were performed in triplicate. All data obtained are expressed as mean of the triplicate ± standard deviation. Statistical significances were determined by Student’s *t*-test or ANOVA. Differences were considered to be significant when *p* < 0.05.

## 3. Results and Discussion

### 3.1. Stability of the Salmon Gelatin and Alginate Mixtures

**Figure 2:**
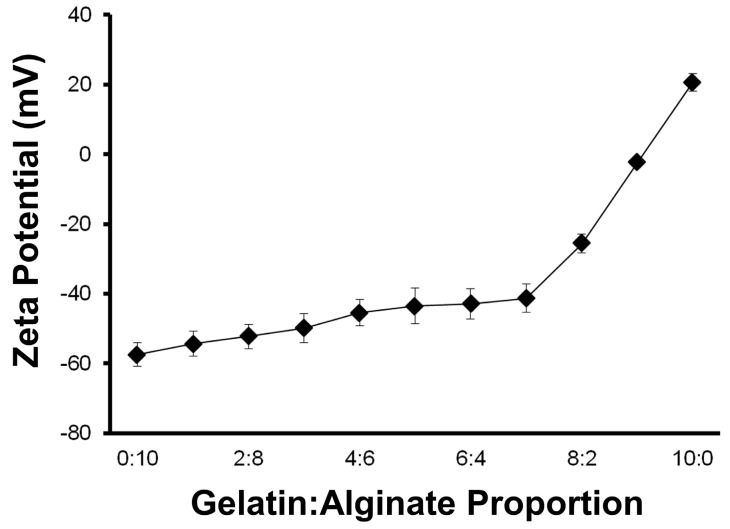
Zeta potential of polymer solutions composed of salmon gelatin and sodium alginate.

The first step to producing these polymer scaffolds was to obtain stable hydrogel precursor solutions without precipitation of the ionic components, salmon gelatin and/or sodium alginate. Conventionally, for colloidal dispersions, Zeta potential values in ranges over |30| mV are considered stable dispersions [30]. The evaluation of the zeta potential of the gelatin and alginate-containing solutions at different ratios showed that high colloidal stability was maintained at gelatin:alginate ratios of <7:3, and more ideally <6:4 (Figure 2).

The stability of the colloidal solution composed of gelatin and alginate was evaluated without pH control. It is well-known that Zeta potential is a function of the pH, but our criterion was to determinate a stable blend point without pH modification, because it reflects the normal fabrication conditions of the scaffolds. However, the pH of the solutions varied only slightly, being 3.6 and 3.2 for mixtures at the extremes of 1:9 and 9:1 (gelatin:alginate), respectively.

Due to their opposing charges, the solutions are expected to form polyelectrolyte complexes when the concentrations of both polymers reach equivalence of their charges [31]. Aqueous solutions containing only alginate exhibited Zeta potential values of ~−60 mV, whereas gelatin was recorded at +20 mV. For the polymer blends evaluated, all resulted in stable dispersions where the negative charge dominated. When the gelatin:alginate ratios were between 4:6 and 6:4, the Zeta potential and pH did not change significantly (*p* > 0.05; ANOVA) and were close to −43 mV and 3.5, respectively. On this basis, the gelatin and alginate were used in equal concentrations for further studies to test the addition of plasticizers and gelling agents.

### 3.2. Scaffold Microstuctures

**Figure 3:**
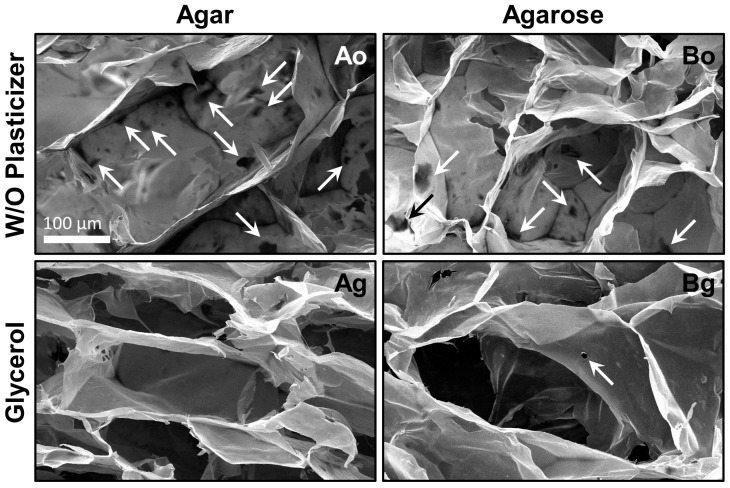
Microstructure of scaffolds (formulations Ao, Ag, Bo, and Bg). Arrows show the micro-holes.

The first scaffolds prepared were made using salmon gelatin and alginate (in equal proportions) with gelling excipient (agar or agarose) and without plasticizer. SEM micrographs of the microstructures obtained when agar (formulation Ao) or agarose (formulation Bo) were used as gelling agents are shown in Figure 3. The materials obtained exhibited unsatisfactory morphologies, with significant defects in the pore walls (micro-holes, arrowed). The average of micro-holes for materials Ao and Bo were close to 4.2 and 3.6 per pore, respectively. This damage could be the result of the freeze-drying processes, since it is known that dry salmon gelatin has crystalline domains [21], and can fracture easily.

In order to improve the structural integrity of the scaffolds, the food grade plasticizer glycerol was added to the formulations. Glycerol is an excellent edible plasticizer used in combination with biopolymers as alginate [32]. Glycerol incorporation served to improve the microstructure and avoid the physical damage during the freeze-drying step. The improved scaffolds due to glycerol addition (formulations Ag and Bg) are shown in Figure 3. Glycerol effectively improved the microstructure of the material, reducing the formation of micro-holes (*p* < 0.05; ANOVA). The average micro-holes in both scaffolds made with glycerol (Ag and Bg) was less than 0.3 per pore. In addition, the pores obtained with glycerol were better defined and had an appropriate size to cultivate cells [15] (see Table 2).

It is known that material biocompatibility is related to the scaffold microstructure [15]. In addition, the addition of glycerol to the material formulation could modify the biocompatibility. Thus, the next step was to evaluate the cell biocompatibility with the scaffolds.

### 3.3. Biocompatibility of the Scaffolds with Myoblasts

**Figure 4:**
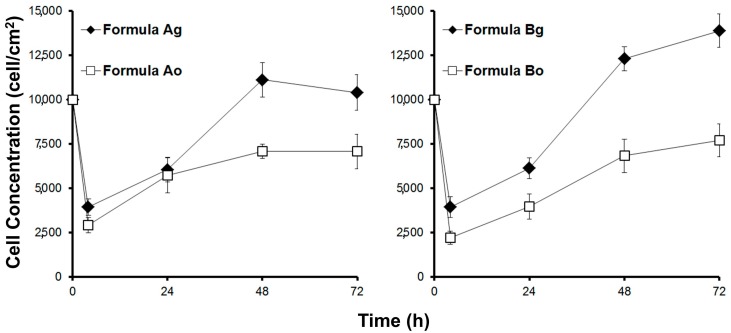
Cell growth of myoblasts seeded onto scaffolds (scaffold thickness ~3 mm).

The biocompatibility was studied by culturing myoblasts in the scaffolds. Cell adhesion and growth of viable cells was measured. Figure 4 shows the viable biomass in the scaffolds over time.

Cell adhesion in the scaffolds made without plasticizer was very low—less than 30% for formulations Ao and Bo. However, the incorporation of the plasticizer glycerol resulted in improved cell adhesion. Close to 40% cell adhesion was obtained in the scaffold with glycerol (shown in Table 2). This result compared with other reports in which a cell adhesion of mesenchymal cells onto biopolymer scaffolds was reported close to 40% [11] as an appropriate value; however, 30% is deemed low.

The growth of myoblasts was markedly more rapid within the scaffold with glycerol, and notably slow in the absence of this plasticizer (see Figure 4). This indicates that the microstructure of the scaffold without plasticizer was not adequate to grow muscle cells. The use of glycerol as a non-toxic plasticizer served to improve scaffold performance. For subsequent studies, only scaffolds containing plasticizers in their formulations were carried through for study. Based on these results, it was decided to perform physical and biological characterizations of both edible scaffolds (formulations Ag and Bg) using the selected plasticizer (glycerol).

### 3.4. Physical Behavior of the Scaffolds

The open structure of the porous scaffold with plasticizer presents a suitable microstructure for cell growth, as described above. The scaffolds containing glycerol and using different gelling agents in their formulation (agar or agarose) had different structures (see Figure 3) and pore size (see Table 2). The pore size when agarose was used as gelling agent was larger than agar (*p* < 0.05; *t*-test), potentially affecting important physical characteristics. For instance, the microstructure may affect the stiffness, which is an important characteristic that can influence muscle cell growth [5,6]. Another aspect that could affect the structural stability and therefore cell compatibility is water–scaffold interaction, and therefore affinity with the cell media surrounding the cells.

**Figure 5:**
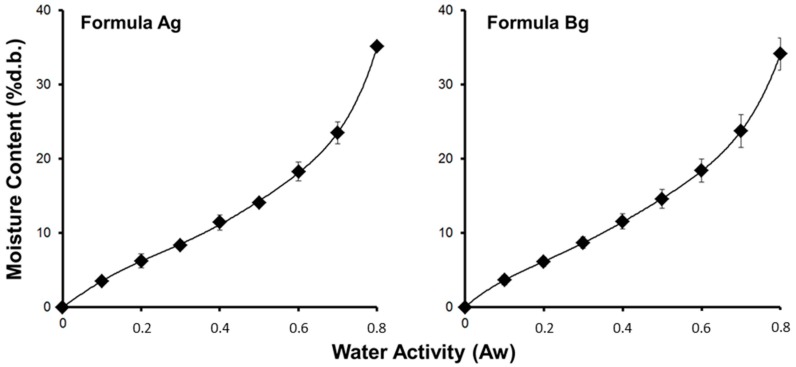
Sorption isotherms of scaffolds.

Sorption isotherms of both scaffolds (Ag and Bg) are shown in Figure 5. The monolayer moisture content calculated from the GAB model is shown in Table 2, and indicates that scaffold formulation Bg was more hygroscopic than scaffold Ag (*p* < 0.05; *t*-test). In addition, the water uptake ratio in the scaffold made with agarose was higher than agar (*p* < 0.05; *t*-test) (see Table 2). These results indicate that the scaffolds made with agarose had better water interaction capacity.

**Figure 6:**
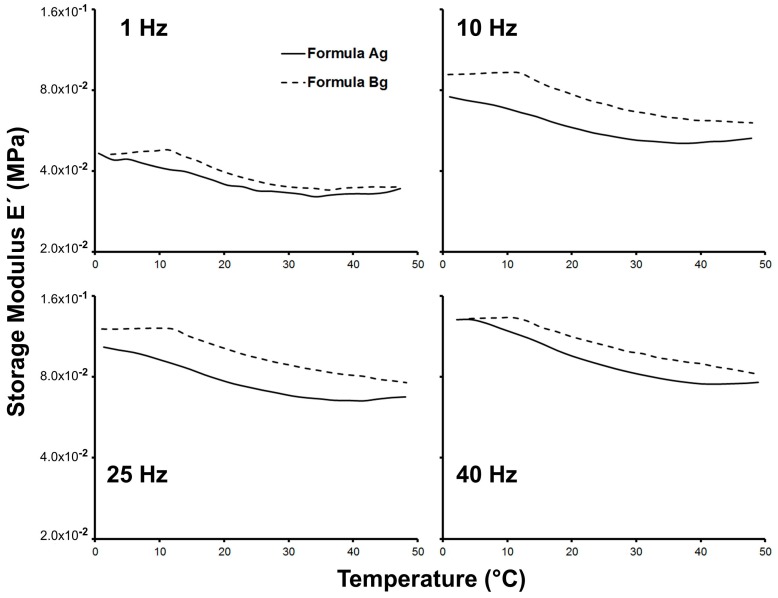
Stiffness of the wet scaffolds.

The mechanical response of the wet scaffolds, reflecting their hydrated properties in cell culture, is summarized in Figure 6. It is very important to note that the wet material is the one in contact with the cells, not the dry material, since the scaffold is immersed in culture media when it is loaded with cells. For this reason, the mechanical properties were measured with the fully hydrated material.

The storage modulus (E’)—which is associated with the stiffness of the material—was determined in the hydrated condition at different frequencies and temperatures, and showed frequency dependency for both Ag and Bg scaffolds. The latter has been reported in hydrogels composed of bovine gelatin and alginate in the same frequency range, which exhibit elastic behavior [33]. Furthermore, it has been reported that stiffness is a physical property that can regulate gene expression, adhesion, and proliferation of the muscle cells [34].

At physiological temperature, close to 37 °C, the stiffness of the scaffold Bg was larger than Ag. This result suggests that interaction between muscle cells and the material should be better when the scaffold is made with agarose.

The results obtained for the physical characterization indicate that agarose is a more appropriate gelling agent for the scaffolds, allowing the formation of a suitable structure, porosity, water interaction, and stiffness.

### 3.5. Biodegradation of the Scaffolds

**Figure 7:**
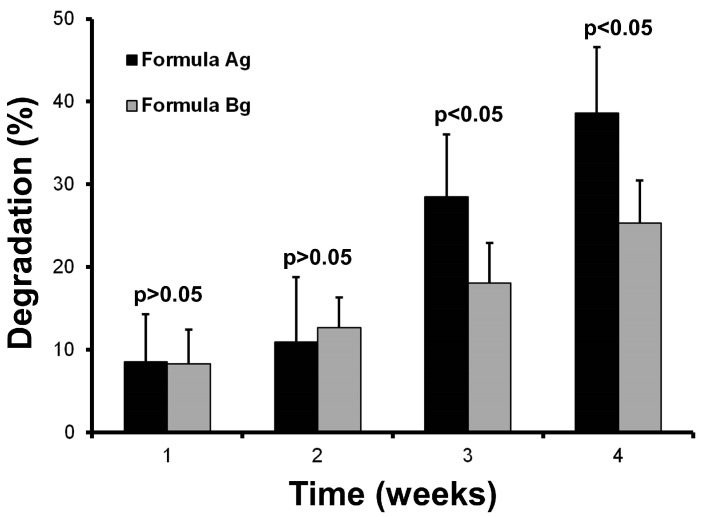
In vitro biodegradation of scaffolds.

The capacity of a material to resist degradation by biological agents is an essential attribute for designing scaffolds that provide long-term conditions and mechanical strength. The degradation produced by lysozymes at 37 °C is a standard assay to compare scaffolds that will be used to culture cells. Figure 7 shows the in vitro biodegradation of the edible scaffolds (formulations Ag and Bg) incubated in simulated physiological conditions.

During the first two weeks, both scaffolds (Ag and Bg) exhibited similar degradation rates (*p* > 0.05; *t*-test). Nevertheless, after the third week, the scaffold made using agar as gelling agent (formulation Ag) showed a high level of degradation. This result indicates that formulation Ag would not be adequate for long-term biological use.

The scaffold produced with agarose as gelling agent (formula Bg) exhibited ~25% biodegradation after four weeks in the presence of lysozyme at 37 °C. This value is comparable with in vitro biodegradation of biopolymer scaffolds designed to regenerate bones [29], indicating a good resistance to be used within bioreactor systems.

These results indicate strongly that agarose is a more suitable gelling agent to preserve the integrity of the scaffold.

### 3.6. Biological Characterization of Myoblasts Cultured in the Scaffolds

**Figure 8:**
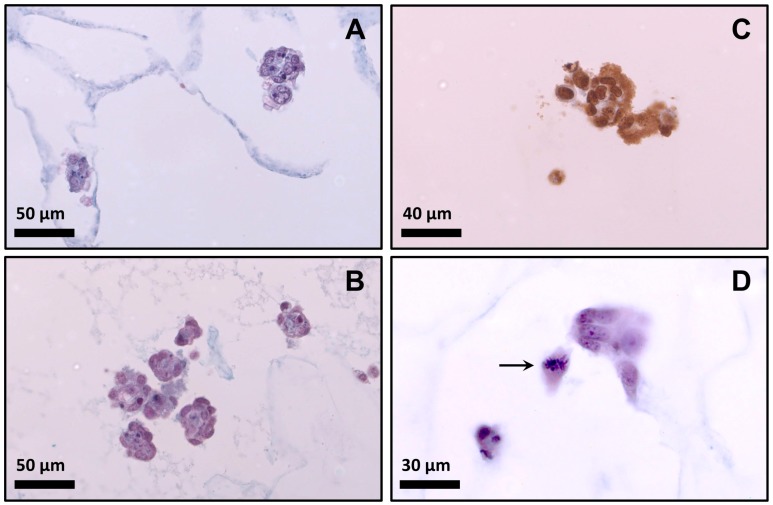
Behavior of myoblasts inside the scaffold after 48 h of seeding. (**A**) Photomicrograph of scaffold Ag (histochemical technique); (**B**) Photomicrograph of scaffold Bg (histochemical technique); (**C**) Cells seeded on scaffold Bg showing positive immunostaining for BrdU (immuno histochemical technique); (**D**) Photomicrograph of scaffold Bg (arrowed cell in metaphase).

The cell morphology of myoblasts cultured in the scaffolds for 48 h is shown in histologically stained sections of Figure 8A,B. It is well known that the morphology of myoblasts in 3D culture differs substantially from those in monolayer culture. Myoblasts did not show fibroblast-like morphology. In early stage of myogenesis, when cells are proliferating within the matrix, they remained rounded, forming later cell–cell connections [13]. After 48 h, these cell–cell contacts were observed as cell clusters (see Figure 8). Other studies have reported the formation of clusters when cells are cultivated in hydrogels [14,35] or scaffolds [11,15], indicating a natural stage of adaptation in the scaffold.

Cell aggregation in clusters is assumed to be an important factor in tissue culture processes. However, from the perspective of mass transfer, cell aggregation in culture generates an additional resistance effect on oxygen transportation, implying local extreme hypoxia at the center, thereby affecting cell viability and function [14,35]. For this reason, it is very important to estimate the cell viability and proliferation inside the scaffolds in order to evaluate if the material affects the behavior of the cells.

The presence of viable cells determined by histochemical analysis was higher in the scaffold made with agarose (*p* < 0.05; *t*-test) (Table 2). The high viability observed for formulation Bg indicates that agarose was superior to agar in this context. It is important to emphasize that formulation Bg had more stiffness compared with scaffold Ag (see Figure 6), which is a relevant parameter for the culture of muscle cells [12,34]. This result, linked with the cell growth kinetic showed in Figure 4, indicates that formulation Bg allowed the cultivation of viable myoblasts. In order to confirm this, formulation Bg was studied using an immunohistochemistry assay to verify the presence of mitotic cells.

Mitotic cells were identified by the incorporation of BrdU into the newly-synthesized DNA of the cells. Figure 8C shows positive reaction to BrdU incorporation in cells cultivated on scaffold Bg, confirming the presence of mitotic cells. This evidence and the high cell growth (see Figure 4) clearly indicate that myoblasts could proliferate in the selected scaffold (formulation Bg). In addition, Figure 8D shows a cell inside the scaffold made with the formula Bg in a clear step of cell division (metaphase).

**Figure 9:**
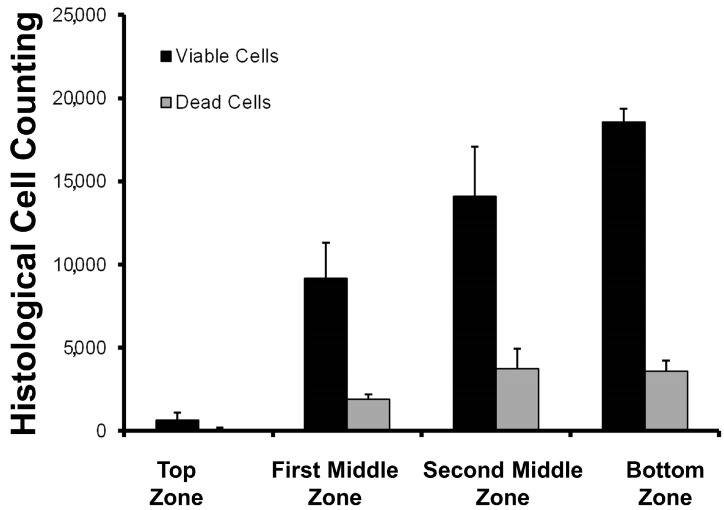
Cell distribution inside the scaffold (formula Bg) after 48 h of seeding. The scaffold thickness is 3 mm (each zone is close to 0.75 mm). The bottom zone is directly in contact with the cell culture plate.

The distribution of the cells inside the selected scaffold (formulation Bg) was not homogeneous (Figure 9). This is an expected result, as cell growth velocity and cell clustering are dependent on nutrients diffusion and oxygen supply [14]. Previous reports have informed us that cell culture into biopolymer blended scaffolds in static culture tend to grow at the bottom of the scaffolds [11]. However, cell viability was high in all scaffold sections. This data is important information for the design of bioreactor systems to model and predict the biomass distribution. In terms of food systems, this parameter could be used to have different layers of muscle fiber across the in vitro meat construct.

The results presented here show that scaffolds made with salmon gelatin, alginate, agarose, and glycerol permit the culture of muscle cells. Additionally, the biological properties of this scaffold allowed an adequate behavior and distribution. Cells seeded into the scaffold could grow, proliferate, and express suitable cell behavior.

## 4. Conclusions

Salmon gelatin can be blended with other non-mammalian biopolymers allowing the preparation of suitable scaffolds to culture myoblasts.

The incorporation of a plasticizer (glycerol) positively modifies the microstructure of the scaffold, changing the pore size and morphology. The biocompatibility with muscle cells (myoblasts)—measured as cell adhesion and cell growth—is strongly dependent on the microstructure the material, as well as material composition. The plasticizer glycerol improves the microstructure and biocompatibility of the scaffolds.

The type of gelling agent used to make the scaffolds affects the pore size and physical properties (stiffness, water uptake, and degradation). The use of agarose instead of agar produces a material with better stiffness, water uptake, and resistance against biodegradation. Myoblast cell growth into the scaffold made with agarose shows adequate cell behavior in terms of viability and proliferation. The gelling agent agarose improves the stiffness and behavior of myoblasts growing into the scaffold.

Scaffolds prepared with non-mammalian biomaterials such as salmon gelatin, alginate, agarose, (gelling agent) and glycerol (plasticizer) can be used as formulations for in vitro meat.

## Figures and Tables

**Table 1:** Formulation of the polymer solutions used for scaffold preparation.

Formulation ^1^	Ao	Ag	Bo	Bg
**Salmon gelatin (% *w*/*v*)**	0.375	0.375	0.375	0.375
**Sodium alginate (% *w*/*v*)**	0.375	0.375	0.375	0.375
**Agar (% *w*/*v*)**	0.250	0.250	-	-
**Agarose (% *w*/*v*)**	-	-	0.250	0.250
**Glycerol (% *w*/*v*)**	-	0.100	-	0.100

^1^ For each formulation name, the letters mean the following: (i) A or B: agar or agarose as gelling excipient, respectively; (ii) g or o: with or without glycerol as plasticizer, respectively.

**Table 2:** Properties of the salmon gelatin/alginate scaffolds made with glycerol as plasticizer.

Properties ^1^	Agar as Gelling (Ag)	Agarose as Gelling (Bg)
**Pore size (µm)**	153.2 (±3.6)	207.8 (±5.4)
**Water uptake ratio (g/g)**	5.8 (±0.5)	12.8 (±1.0)
**Monolayer moisture content (% d.b.)**	8.6 (±0.5)	9.4 (±0.1)
**Cell adhesion (%)**	39.4 (±4.6)	39.5 (±5.8)
**Cell duplication time (h)**	27.4 (±2.1)	23.9 (±1.8)
**Cell viability (%)**	69.2 (±5.5)	85.1 (±2.9)

^1^ Properties values are expressed as mean ± standard deviation.
